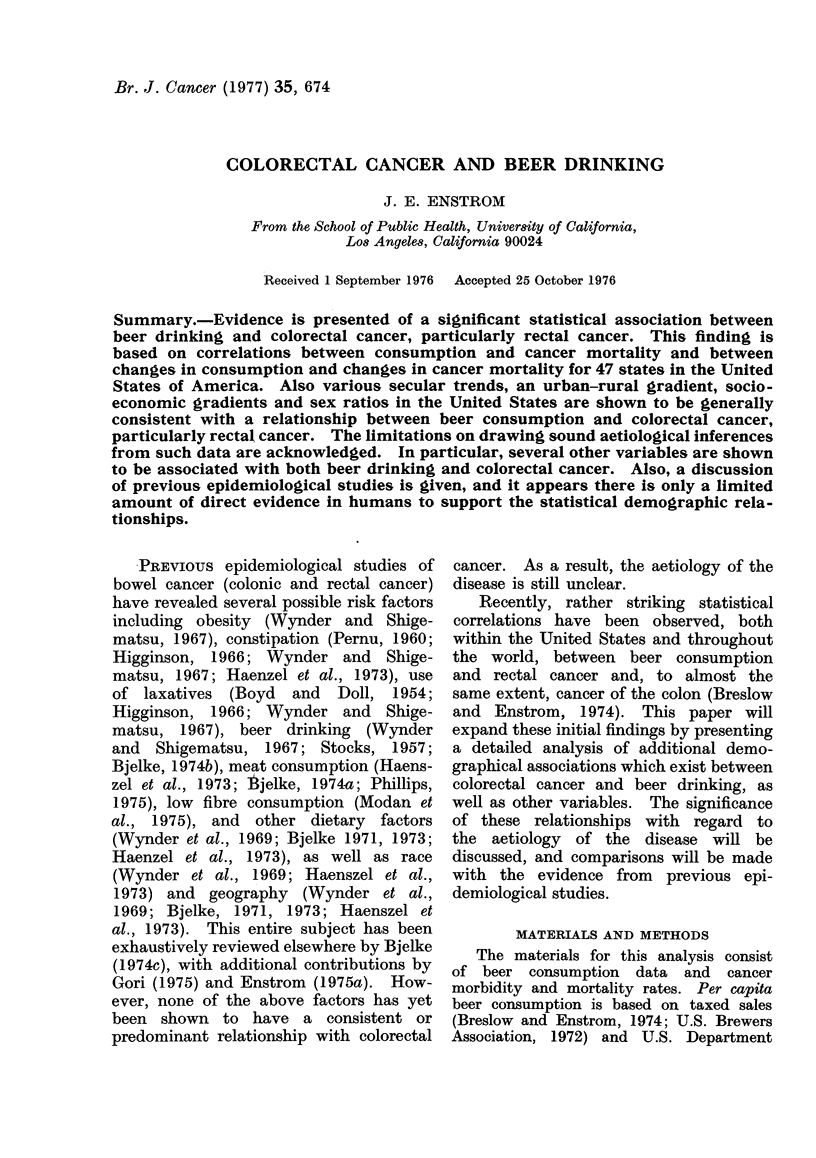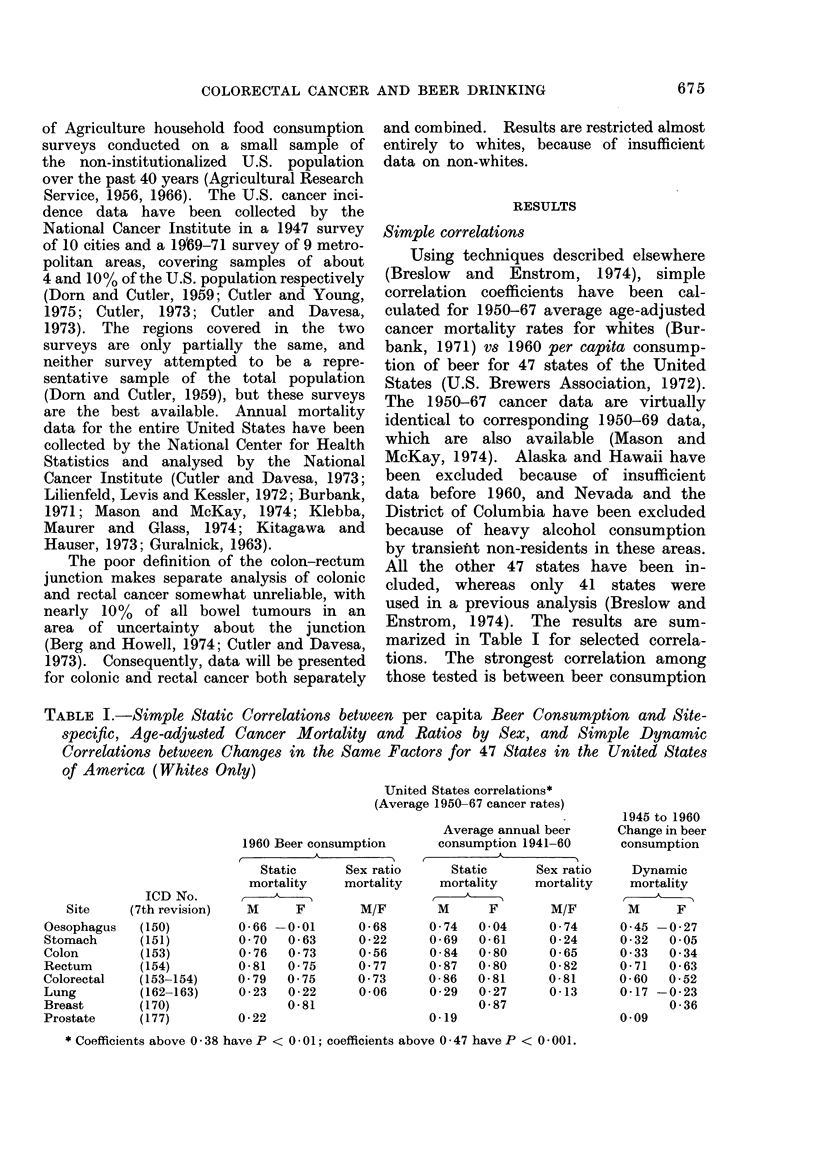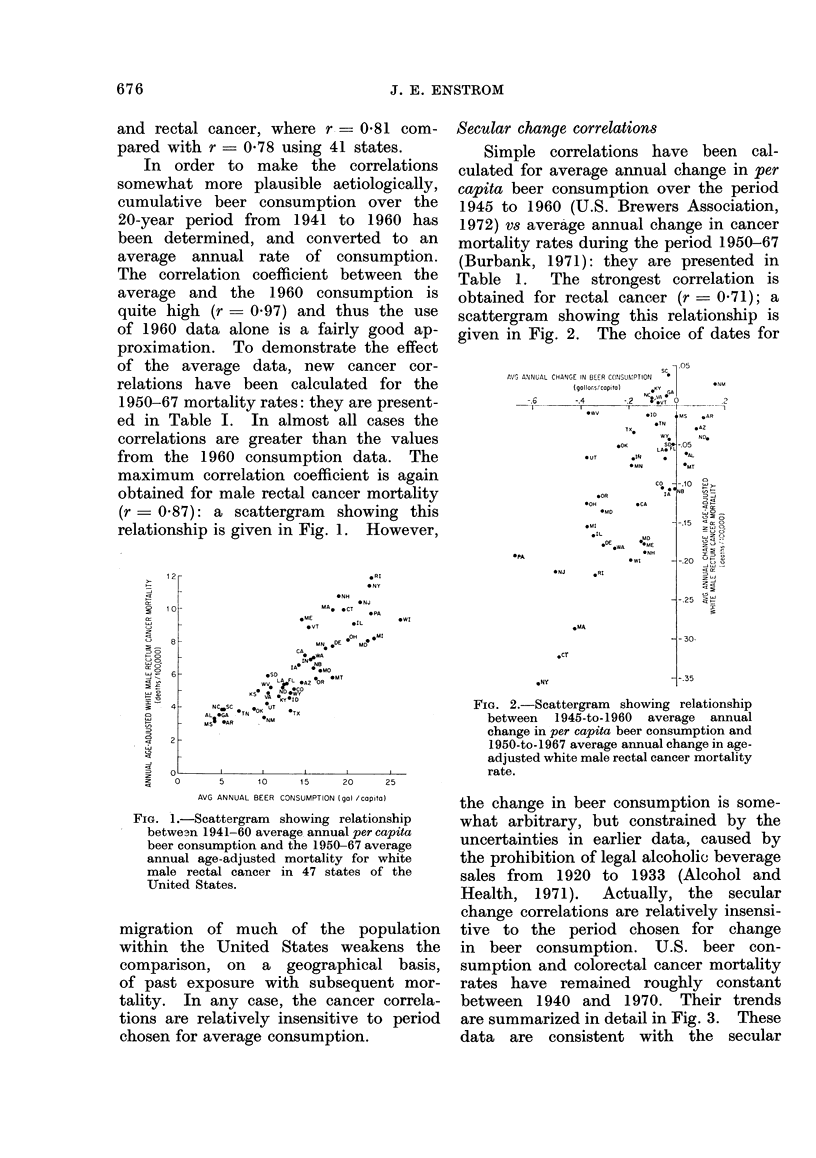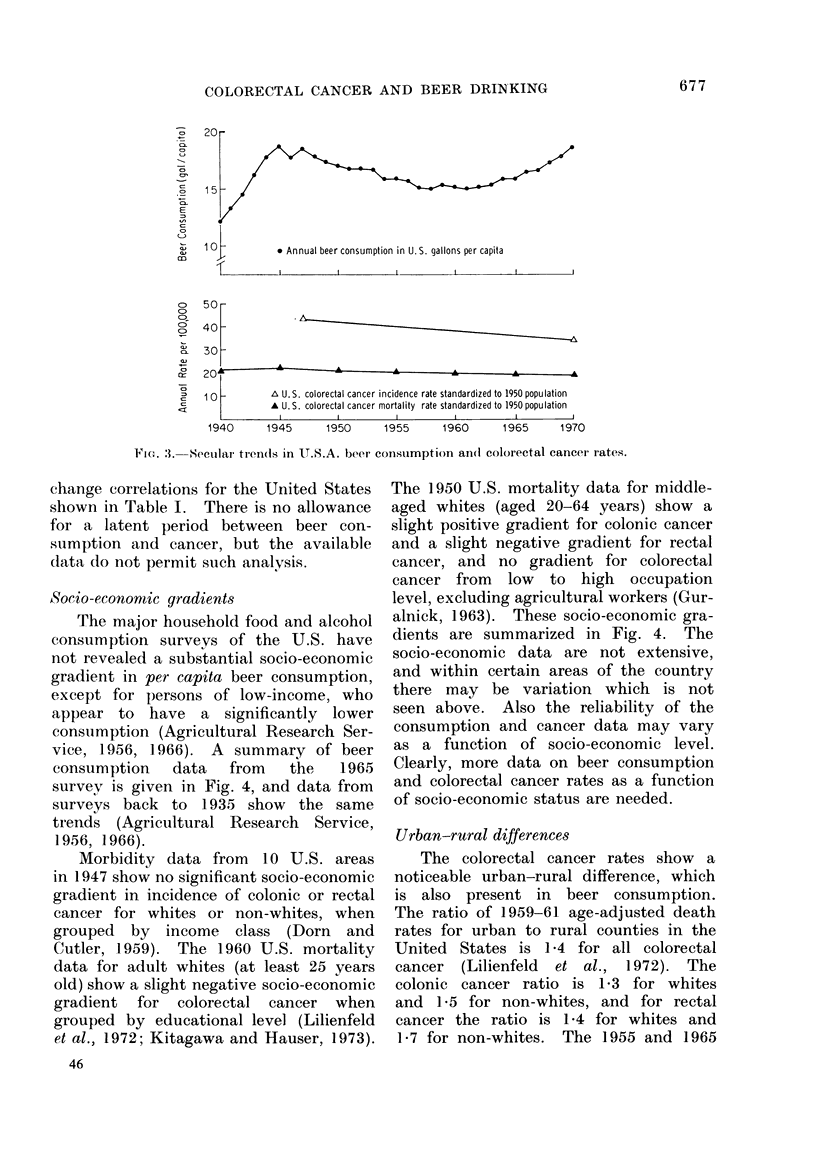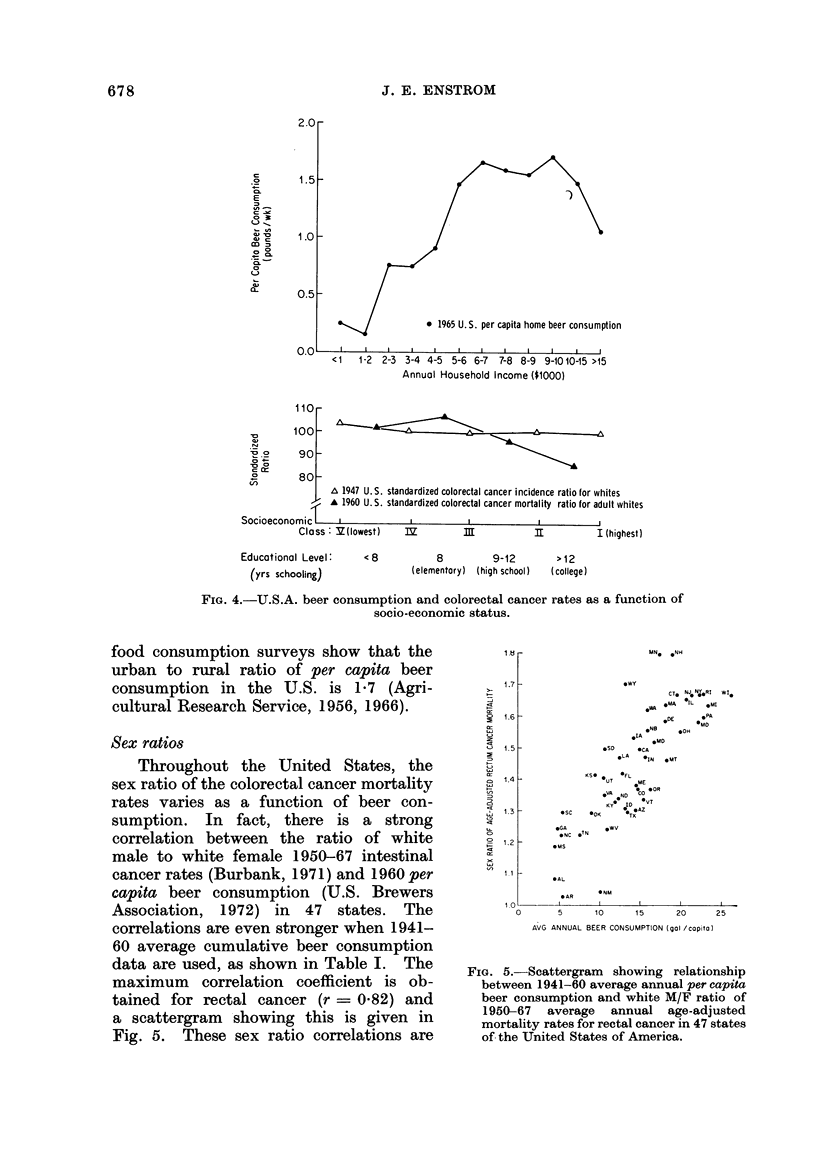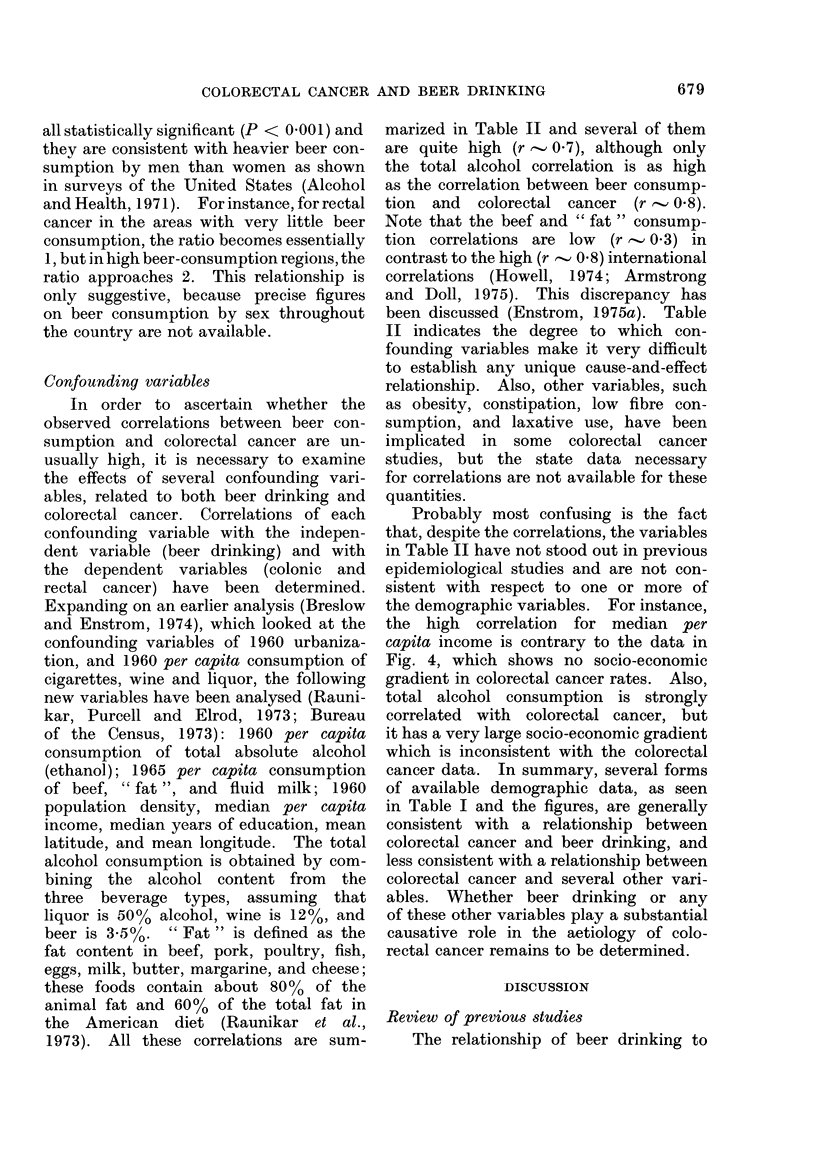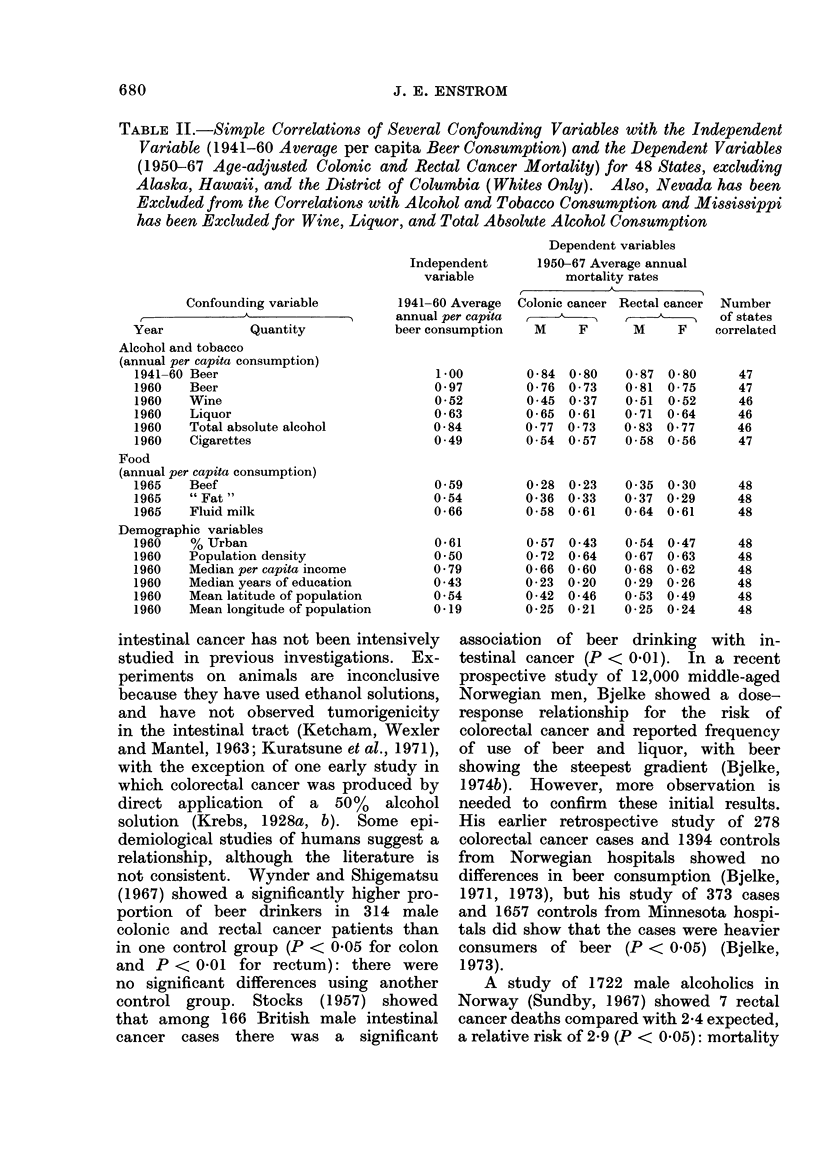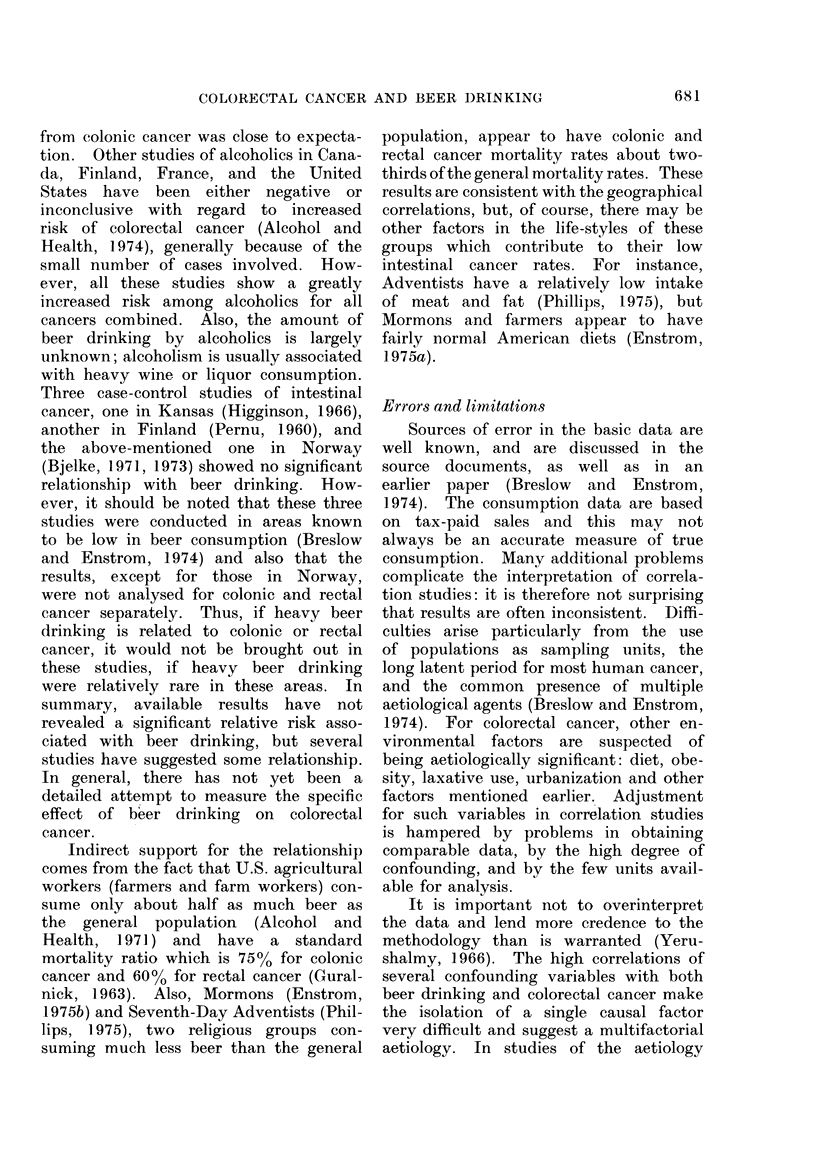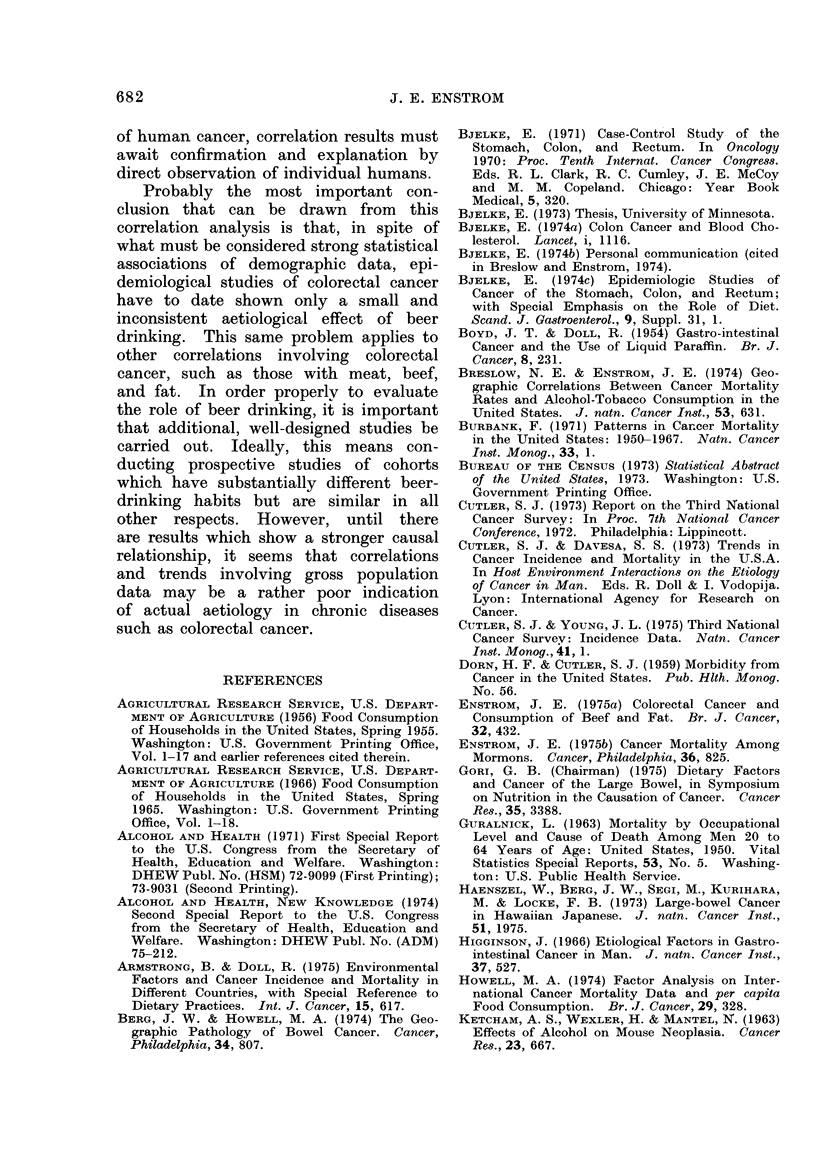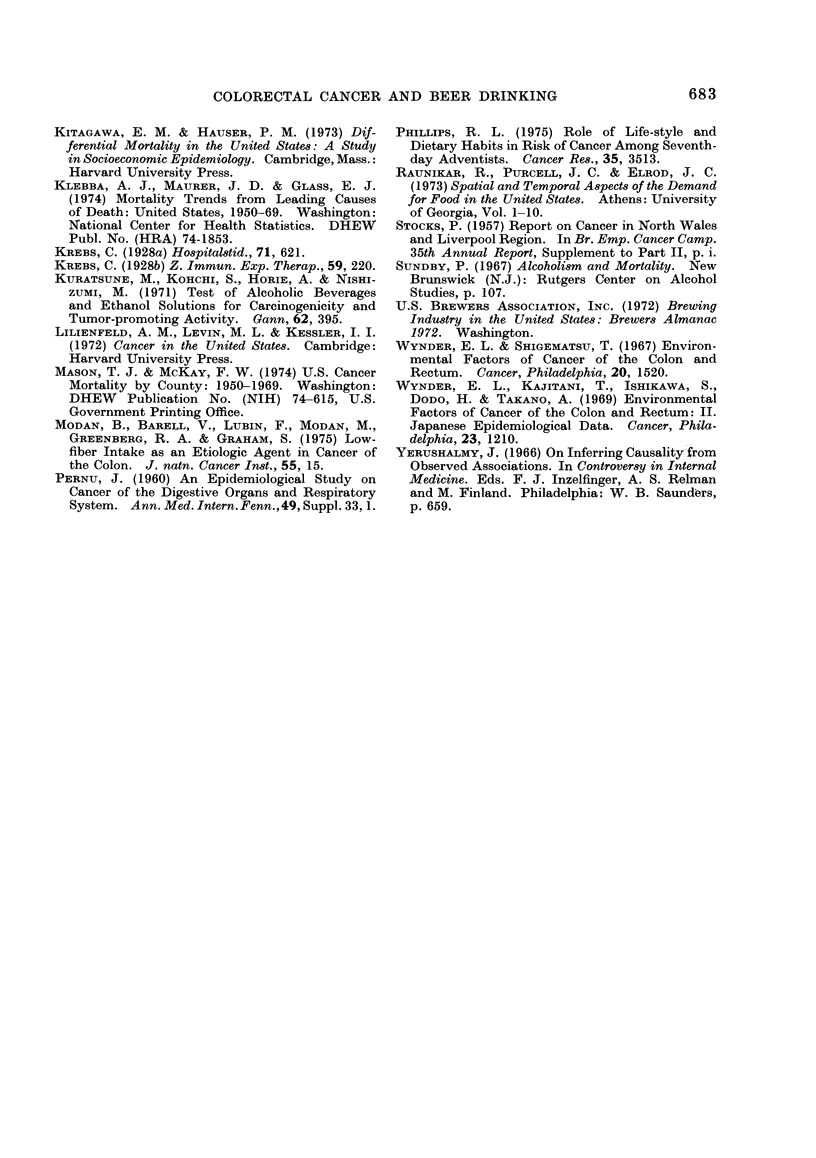# Colorectal cancer and beer drinking.

**DOI:** 10.1038/bjc.1977.103

**Published:** 1977-05

**Authors:** J. E. Enstrom

## Abstract

Evidence is presented of a significant statistical association between beer drinking and colorectal cancer, particularly rectal cancer. This finding is based on correlations between consumption and cancer mortality and between changes in consumption and changes in cancer mortality for 47 states in the United States of America. Also various secular trends, an urban-rural gradient, socioeconomic gradients and sex ratios in the United States are shown to be generally consistent with a relationship between beer consumption and colorectal cancer, particularly rectal cancer. The limitations on drawing sound aetiological inferences from such data are acknowledged. In particular, several other variables are shown to be associated with both beer drinking and colorectal cancer. Also, a discussion of previous epidemiological studies is given, and it appears there is only a limited amount of direct evidence in humans to support the statistical demographic relationships.


*Br. J. Cancer* (1977) **35**, 674

### COLORECTAL CANCER AND BEER DRINKING

J. E. ENSTROM

*From the School of Public Health, University of California, Los Angeles, California* 90024

Received 1 September 1976  Accepted 25 October 1976

**Summary.—Evidence is presented of a significant statistical association between beer drinking and colorectal cancer, particularly rectal cancer. This finding is based on correlations between consumption and cancer mortality and between changes in consumption and changes in cancer mortality for 47 states in the United States of America. Also various secular trends, an urban–rural gradient, socio-economic gradients and sex ratios in the United States are shown to be generally consistent with a relationship between beer consumption and colorectal cancer, particularly rectal cancer. The limitations on drawing sound aetiological inferences from such data are acknowledged. In particular, several other variables are shown to be associated with both beer drinking and colorectal cancer. Also, a discussion of previous epidemiological studies is given, and it appears there is only a limited amount of direct evidence in humans to support the statistical demographic relationships.**

PREVIOUS epidemiological studies of bowel cancer (colonic and rectal cancer) have revealed several possible risk factors including obesity (Wynder and Shigematsu, 1967), constipation (Pernu, 1960; Higginson, 1966; Wynder and Shigematsu, 1967; Haenzel *et al.*, 1973), use of laxatives (Boyd and Doll, 1954; Higginson, 1966; Wynder and Shigematsu, 1967), beer drinking (Wynder and Shigematsu, 1967; Stocks, 1957; Bjelke, 1974*b*), meat consumption (Haenszel *et al.*, 1973; Bjelke, 1974*a*; Phillips, 1975), low fibre consumption (Modan *et al.*, 1975), and other dietary factors (Wynder *et al.*, 1969; Bjelke 1971, 1973; Haenzel *et al.*, 1973), as well as race (Wynder *et al.*, 1969; Haenszel *et al.*, 1973) and geography (Wynder *et al.*, 1969; Bjelke, 1971, 1973; Haenszel *et al.*, 1973). This entire subject has been exhaustively reviewed elsewhere by Bjelke (1974*c*), with additional contributions by Gori (1975) and Enstrom (1975*a*). However, none of the above factors has yet been shown to have a consistent or predominant relationship with colorectal cancer. As a result, the aetiology of the disease is still unclear.

Recently, rather striking statistical correlations have been observed, both within the United States and throughout the world, between beer consumption and rectal cancer and, to almost the same extent, cancer of the colon (Breslow and Enstrom, 1974). This paper will expand these initial findings by presenting a detailed analysis of additional demographical associations which exist between colorectal cancer and beer drinking, as well as other variables. The significance of these relationships with regard to the aetiology of the disease will be discussed, and comparisons will be made with the evidence from previous epidemiological studies.

### MATERIALS AND METHODS

The materials for this analysis consist of beer consumption data and cancer morbidity and mortality rates. *Per capita* beer consumption is based on taxed sales (Breslow and Enstrom, 1974; U.S. Brewers Association, 1972) and U.S. Department of Agriculture household food consumption surveys conducted on a small sample of the non-institutionalized U.S. population over the past 40 years (Agricultural Research Service, 1956, 1966). The U.S. cancer incidence data have been collected by the National Cancer Institute in a 1947 survey of 10 cities and a 1969–71 survey of 9 metropolitan areas, covering samples of about 4 and 10% of the U.S. population respectively (Dorn and Cutler, 1959; Cutler and Young, 1975; Cutler, 1973; Cutler and Davesa, 1973). The regions covered in the two surveys are only partially the same, and neither survey attempted to be a representative sample of the total population (Dorn and Cutler, 1959), but these surveys are the best available. Annual mortality data for the entire United States have been collected by the National Center for Health Statistics and analysed by the National Cancer Institute (Cutler and Davesa, 1973; Lilienfeld, Levis and Kessler, 1972; Burbank, 1971; Mason and McKay, 1974; Klebba, Maurer and Glass, 1974; Kitagawa and Hauser, 1973; Guralnick, 1963).

The poor definition of the colon–rectum junction makes separate analysis of colonic and rectal cancer somewhat unreliable, with nearly 10% of all bowel tumours in an area of uncertainty about the junction (Berg and Howell, 1974; Cutler and Davesa, 1973). Consequently, data will be presented for colonic and rectal cancer both separately and combined. Results are restricted almost entirely to whites, because of insufficient data on non-whites.

### RESULTS

### *Simple correlations*

Using techniques described elsewhere (Breslow and Enstrom, 1974), simple correlation coefficients have been calculated for 1950–67 average age-adjusted cancer mortality rates for whites (Burbank, 1971) *vs* 1960 *per capita* consumption of beer for 47 states of the United States (U.S. Brewers Association, 1972). The 1950–67 cancer data are virtually identical to corresponding 1950–69 data, which are also available (Mason and McKay, 1974). Alaska and Hawaii have been excluded because of insufficient data before 1960, and Nevada and the District of Columbia have been excluded because of heavy alcohol consumption by transient non-residents in these areas. All the other 47 states have been included, whereas only 41 states were used in a previous analysis (Breslow and Enstrom, 1974). The results are summarized in Table I for selected correlations. The strongest correlation among those tested is between beer consumption

TABLE I.—*Simple Static Correlations between* per capita *Beer Consumption and Site-specific, Age-adjusted Cancer Mortality and Ratios by Sex, and Simple Dynamic Correlations between Changes in the Same Factors for* 47 *States in the United States of America* (*Whites Only*)

| | | United States correlations* (Average 1950–67 cancer rates) | | | | | | 1945 to 1960 Change in beer consumption | |
|---|---|---|---|---|---|---|---|---|---|
| | | 1960 Beer consumption | | | Average annual beer consumption 1941–60 | | | | |
| | | Static mortality | | Sex ratio mortality | Static mortality | | Sex ratio mortality | Dynamic mortality | |
| Site | ICD No. (7th revision) | M | F | M/F | M | F | M/F | M | F |
| Oesophagus | (150) | 0·66 | −0·01 | 0·68 | 0·74 | 0·04 | 0·74 | 0·45 | −0·27 |
| Stomach | (151) | 0·70 | 0·63 | 0·22 | 0·69 | 0·61 | 0·24 | 0·32 | 0·05 |
| Colon | (153) | 0·76 | 0·73 | 0·56 | 0·84 | 0·80 | 0·65 | 0·33 | 0·34 |
| Rectum | (154) | 0·81 | 0·75 | 0·77 | 0·87 | 0·80 | 0·82 | 0·71 | 0·63 |
| Colorectal | (153–154) | 0·79 | 0·75 | 0·73 | 0·86 | 0·81 | 0·81 | 0·60 | 0·52 |
| Lung | (162–163) | 0·23 | 0·22 | 0·06 | 0·29 | 0·27 | 0·13 | 0·17 | −0·23 |
| Breast | (170) | | 0·81 | | | 0·87 | | | 0·36 |
| Prostate | (177) | 0·22 | | | 0·19 | | | 0·09 | |

* Coefficients above 0·38 have $P < 0\cdot01$; coefficients above 0·47 have $P < 0\cdot001$.

and rectal cancer, where $r = 0{\cdot}81$ compared with $r = 0{\cdot}78$ using 41 states.

In order to make the correlations somewhat more plausible aetiologically, cumulative beer consumption over the 20-year period from 1941 to 1960 has been determined, and converted to an average annual rate of consumption. The correlation coefficient between the average and the 1960 consumption is quite high ($r = 0{\cdot}97$) and thus the use of 1960 data alone is a fairly good approximation. To demonstrate the effect of the average data, new cancer correlations have been calculated for the 1950–67 mortality rates: they are presented in Table I. In almost all cases the correlations are greater than the values from the 1960 consumption data. The maximum correlation coefficient is again obtained for male rectal cancer mortality ($r = 0{\cdot}87$): a scattergram showing this relationship is given in Fig. 1. However,

FIG. 1.—Scattergram showing relationship between 1941–60 average annual *per capita* beer consumption and the 1950–67 average annual age-adjusted mortality for white male rectal cancer in 47 states of the United States.

migration of much of the population within the United States weakens the comparison, on a geographical basis, of past exposure with subsequent mortality. In any case, the cancer correlations are relatively insensitive to period chosen for average consumption.

*Secular change correlations*

Simple correlations have been calculated for average annual change in *per capita* beer consumption over the period 1945 to 1960 (U.S. Brewers Association, 1972) *vs* average annual change in cancer mortality rates during the period 1950–67 (Burbank, 1971): they are presented in Table 1. The strongest correlation is obtained for rectal cancer ($r = 0{\cdot}71$); a scattergram showing this relationship is given in Fig. 2. The choice of dates for

FIG. 2.—Scattergram showing relationship between 1945-to-1960 average annual change in *per capita* beer consumption and 1950-to-1967 average annual change in age-adjusted white male rectal cancer mortality rate.

the change in beer consumption is somewhat arbitrary, but constrained by the uncertainties in earlier data, caused by the prohibition of legal alcoholic beverage sales from 1920 to 1933 (Alcohol and Health, 1971). Actually, the secular change correlations are relatively insensitive to the period chosen for change in beer consumption. U.S. beer consumption and colorectal cancer mortality rates have remained roughly constant between 1940 and 1970. Their trends are summarized in detail in Fig. 3. These data are consistent with the secular

FIG. 3.—Secular trends in U.S.A. beer consumption and colorectal cancer rates.

change correlations for the United States shown in Table I. There is no allowance for a latent period between beer consumption and cancer, but the available data do not permit such analysis.

*Socio-economic gradients*

The major household food and alcohol consumption surveys of the U.S. have not revealed a substantial socio-economic gradient in *per capita* beer consumption, except for persons of low-income, who appear to have a significantly lower consumption (Agricultural Research Service, 1956, 1966). A summary of beer consumption data from the 1965 survey is given in Fig. 4, and data from surveys back to 1935 show the same trends (Agricultural Research Service, 1956, 1966).

Morbidity data from 10 U.S. areas in 1947 show no significant socio-economic gradient in incidence of colonic or rectal cancer for whites or non-whites, when grouped by income class (Dorn and Cutler, 1959). The 1960 U.S. mortality data for adult whites (at least 25 years old) show a slight negative socio-economic gradient for colorectal cancer when grouped by educational level (Lilienfeld *et al.*, 1972; Kitagawa and Hauser, 1973). The 1950 U.S. mortality data for middle-aged whites (aged 20–64 years) show a slight positive gradient for colonic cancer and a slight negative gradient for rectal cancer, and no gradient for colorectal cancer from low to high occupation level, excluding agricultural workers (Guralnick, 1963). These socio-economic gradients are summarized in Fig. 4. The socio-economic data are not extensive, and within certain areas of the country there may be variation which is not seen above. Also the reliability of the consumption and cancer data may vary as a function of socio-economic level. Clearly, more data on beer consumption and colorectal cancer rates as a function of socio-economic status are needed.

*Urban–rural differences*

The colorectal cancer rates show a noticeable urban–rural difference, which is also present in beer consumption. The ratio of 1959–61 age-adjusted death rates for urban to rural counties in the United States is 1·4 for all colorectal cancer (Lilienfeld *et al.*, 1972). The colonic cancer ratio is 1·3 for whites and 1·5 for non-whites, and for rectal cancer the ratio is 1·4 for whites and 1·7 for non-whites. The 1955 and 1965

Fig. 4.—U.S.A. beer consumption and colorectal cancer rates as a function of socio-economic status.

food consumption surveys show that the urban to rural ratio of *per capita* beer consumption in the U.S. is 1·7 (Agricultural Research Service, 1956, 1966).

### *Sex ratios*

Throughout the United States, the sex ratio of the colorectal cancer mortality rates varies as a function of beer consumption. In fact, there is a strong correlation between the ratio of white male to white female 1950–67 intestinal cancer rates (Burbank, 1971) and 1960 *per capita* beer consumption (U.S. Brewers Association, 1972) in 47 states. The correlations are even stronger when 1941–60 average cumulative beer consumption data are used, as shown in Table I. The maximum correlation coefficient is obtained for rectal cancer ($r = 0{\cdot}82$) and a scattergram showing this is given in Fig. 5. These sex ratio correlations are

Fig. 5.—Scattergram showing relationship between 1941–60 average annual *per capita* beer consumption and white M/F ratio of 1950–67 average annual age-adjusted mortality rates for rectal cancer in 47 states of the United States of America.

all statistically significant ($P < 0{\cdot}001$) and they are consistent with heavier beer consumption by men than women as shown in surveys of the United States (Alcohol and Health, 1971). For instance, for rectal cancer in the areas with very little beer consumption, the ratio becomes essentially 1, but in high beer-consumption regions, the ratio approaches 2. This relationship is only suggestive, because precise figures on beer consumption by sex throughout the country are not available.

*Confounding variables*

In order to ascertain whether the observed correlations between beer consumption and colorectal cancer are unusually high, it is necessary to examine the effects of several confounding variables, related to both beer drinking and colorectal cancer. Correlations of each confounding variable with the independent variable (beer drinking) and with the dependent variables (colonic and rectal cancer) have been determined. Expanding on an earlier analysis (Breslow and Enstrom, 1974), which looked at the confounding variables of 1960 urbanization, and 1960 *per capita* consumption of cigarettes, wine and liquor, the following new variables have been analysed (Raunikar, Purcell and Elrod, 1973; Bureau of the Census, 1973): 1960 *per capita* consumption of total absolute alcohol (ethanol); 1965 *per capita* consumption of beef, "fat", and fluid milk; 1960 population density, median *per capita* income, median years of education, mean latitude, and mean longitude. The total alcohol consumption is obtained by combining the alcohol content from the three beverage types, assuming that liquor is 50% alcohol, wine is 12%, and beer is 3·5%. "Fat" is defined as the fat content in beef, pork, poultry, fish, eggs, milk, butter, margarine, and cheese; these foods contain about 80% of the animal fat and 60% of the total fat in the American diet (Raunikar *et al.*, 1973). All these correlations are summarized in Table II and several of them are quite high ($r \sim 0{\cdot}7$), although only the total alcohol correlation is as high as the correlation between beer consumption and colorectal cancer ($r \sim 0{\cdot}8$). Note that the beef and "fat" consumption correlations are low ($r \sim 0{\cdot}3$) in contrast to the high ($r \sim 0{\cdot}8$) international correlations (Howell, 1974; Armstrong and Doll, 1975). This discrepancy has been discussed (Enstrom, 1975*a*). Table II indicates the degree to which confounding variables make it very difficult to establish any unique cause-and-effect relationship. Also, other variables, such as obesity, constipation, low fibre consumption, and laxative use, have been implicated in some colorectal cancer studies, but the state data necessary for correlations are not available for these quantities.

Probably most confusing is the fact that, despite the correlations, the variables in Table II have not stood out in previous epidemiological studies and are not consistent with respect to one or more of the demographic variables. For instance, the high correlation for median *per capita* income is contrary to the data in Fig. 4, which shows no socio-economic gradient in colorectal cancer rates. Also, total alcohol consumption is strongly correlated with colorectal cancer, but it has a very large socio-economic gradient which is inconsistent with the colorectal cancer data. In summary, several forms of available demographic data, as seen in Table I and the figures, are generally consistent with a relationship between colorectal cancer and beer drinking, and less consistent with a relationship between colorectal cancer and several other variables. Whether beer drinking or any of these other variables play a substantial causative role in the aetiology of colorectal cancer remains to be determined.

### DISCUSSION

*Review of previous studies*

The relationship of beer drinking to

TABLE II.—*Simple Correlations of Several Confounding Variables with the Independent Variable* (1941–60 *Average* per capita *Beer Consumption*) *and the Dependent Variables* (1950–67 *Age-adjusted Colonic and Rectal Cancer Mortality*) *for* 48 *States, excluding Alaska, Hawaii, and the District of Columbia* (*Whites Only*). *Also, Nevada has been Excluded from the Correlations with Alcohol and Tobacco Consumption and Mississippi has been Excluded for Wine, Liquor, and Total Absolute Alcohol Consumption*

| Confounding variable | | Independent variable | Dependent variables: 1950–67 Average annual mortality rates | | | | |
|---|---|---|---|---|---|---|---|
| | | 1941–60 Average annual *per capita* beer consumption | Colonic cancer | | Rectal cancer | | Number of states correlated |
| Year | Quantity | | M | F | M | F | |
| Alcohol and tobacco (annual *per capita* consumption) | | | | | | | |
| 1941–60 | Beer | 1·00 | 0·84 | 0·80 | 0·87 | 0·80 | 47 |
| 1960 | Beer | 0·97 | 0·76 | 0·73 | 0·81 | 0·75 | 47 |
| 1960 | Wine | 0·52 | 0·45 | 0·37 | 0·51 | 0·52 | 46 |
| 1960 | Liquor | 0·63 | 0·65 | 0·61 | 0·71 | 0·64 | 46 |
| 1960 | Total absolute alcohol | 0·84 | 0·77 | 0·73 | 0·83 | 0·77 | 46 |
| 1960 | Cigarettes | 0·49 | 0·54 | 0·57 | 0·58 | 0·56 | 47 |
| Food (annual *per capita* consumption) | | | | | | | |
| 1965 | Beef | 0·59 | 0·28 | 0·23 | 0·35 | 0·30 | 48 |
| 1965 | "Fat" | 0·54 | 0·36 | 0·33 | 0·37 | 0·29 | 48 |
| 1965 | Fluid milk | 0·66 | 0·58 | 0·61 | 0·64 | 0·61 | 48 |
| Demographic variables | | | | | | | |
| 1960 | % Urban | 0·61 | 0·57 | 0·43 | 0·54 | 0·47 | 48 |
| 1960 | Population density | 0·50 | 0·72 | 0·64 | 0·67 | 0·63 | 48 |
| 1960 | Median *per capita* income | 0·79 | 0·66 | 0·60 | 0·68 | 0·62 | 48 |
| 1960 | Median years of education | 0·43 | 0·23 | 0·20 | 0·29 | 0·26 | 48 |
| 1960 | Mean latitude of population | 0·54 | 0·42 | 0·46 | 0·53 | 0·49 | 48 |
| 1960 | Mean longitude of population | 0·19 | 0·25 | 0·21 | 0·25 | 0·24 | 48 |

intestinal cancer has not been intensively studied in previous investigations. Experiments on animals are inconclusive because they have used ethanol solutions, and have not observed tumorigenicity in the intestinal tract (Ketcham, Wexler and Mantel, 1963; Kuratsune *et al.*, 1971), with the exception of one early study in which colorectal cancer was produced by direct application of a 50% alcohol solution (Krebs, 1928*a*, *b*). Some epidemiological studies of humans suggest a relationship, although the literature is not consistent. Wynder and Shigematsu (1967) showed a significantly higher proportion of beer drinkers in 314 male colonic and rectal cancer patients than in one control group ($P < 0{\cdot}05$ for colon and $P < 0{\cdot}01$ for rectum): there were no significant differences using another control group. Stocks (1957) showed that among 166 British male intestinal cancer cases there was a significant association of beer drinking with intestinal cancer ($P < 0{\cdot}01$). In a recent prospective study of 12,000 middle-aged Norwegian men, Bjelke showed a dose–response relationship for the risk of colorectal cancer and reported frequency of use of beer and liquor, with beer showing the steepest gradient (Bjelke, 1974*b*). However, more observation is needed to confirm these initial results. His earlier retrospective study of 278 colorectal cancer cases and 1394 controls from Norwegian hospitals showed no differences in beer consumption (Bjelke, 1971, 1973), but his study of 373 cases and 1657 controls from Minnesota hospitals did show that the cases were heavier consumers of beer ($P < 0{\cdot}05$) (Bjelke, 1973).

A study of 1722 male alcoholics in Norway (Sundby, 1967) showed 7 rectal cancer deaths compared with 2·4 expected, a relative risk of 2·9 ($P < 0{\cdot}05$): mortality from colonic cancer was close to expectation. Other studies of alcoholics in Canada, Finland, France, and the United States have been either negative or inconclusive with regard to increased risk of colorectal cancer (Alcohol and Health, 1974), generally because of the small number of cases involved. However, all these studies show a greatly increased risk among alcoholics for all cancers combined. Also, the amount of beer drinking by alcoholics is largely unknown; alcoholism is usually associated with heavy wine or liquor consumption. Three case-control studies of intestinal cancer, one in Kansas (Higginson, 1966), another in Finland (Pernu, 1960), and the above-mentioned one in Norway (Bjelke, 1971, 1973) showed no significant relationship with beer drinking. However, it should be noted that these three studies were conducted in areas known to be low in beer consumption (Breslow and Enstrom, 1974) and also that the results, except for those in Norway, were not analysed for colonic and rectal cancer separately. Thus, if heavy beer drinking is related to colonic or rectal cancer, it would not be brought out in these studies, if heavy beer drinking were relatively rare in these areas. In summary, available results have not revealed a significant relative risk associated with beer drinking, but several studies have suggested some relationship. In general, there has not yet been a detailed attempt to measure the specific effect of beer drinking on colorectal cancer.

Indirect support for the relationship comes from the fact that U.S. agricultural workers (farmers and farm workers) consume only about half as much beer as the general population (Alcohol and Health, 1971) and have a standard mortality ratio which is 75% for colonic cancer and 60% for rectal cancer (Guralnick, 1963). Also, Mormons (Enstrom, 1975*b*) and Seventh-Day Adventists (Phillips, 1975), two religious groups consuming much less beer than the general population, appear to have colonic and rectal cancer mortality rates about two-thirds of the general mortality rates. These results are consistent with the geographical correlations, but, of course, there may be other factors in the life-styles of these groups which contribute to their low intestinal cancer rates. For instance, Adventists have a relatively low intake of meat and fat (Phillips, 1975), but Mormons and farmers appear to have fairly normal American diets (Enstrom, 1975*a*).

### *Errors and limitations*

Sources of error in the basic data are well known, and are discussed in the source documents, as well as in an earlier paper (Breslow and Enstrom, 1974). The consumption data are based on tax-paid sales and this may not always be an accurate measure of true consumption. Many additional problems complicate the interpretation of correlation studies: it is therefore not surprising that results are often inconsistent. Difficulties arise particularly from the use of populations as sampling units, the long latent period for most human cancer, and the common presence of multiple aetiological agents (Breslow and Enstrom, 1974). For colorectal cancer, other environmental factors are suspected of being aetiologically significant: diet, obesity, laxative use, urbanization and other factors mentioned earlier. Adjustment for such variables in correlation studies is hampered by problems in obtaining comparable data, by the high degree of confounding, and by the few units available for analysis.

It is important not to overinterpret the data and lend more credence to the methodology than is warranted (Yerushalmy, 1966). The high correlations of several confounding variables with both beer drinking and colorectal cancer make the isolation of a single causal factor very difficult and suggest a multifactorial aetiology. In studies of the aetiology of human cancer, correlation results must await confirmation and explanation by direct observation of individual humans.

Probably the most important conclusion that can be drawn from this correlation analysis is that, in spite of what must be considered strong statistical associations of demographic data, epidemiological studies of colorectal cancer have to date shown only a small and inconsistent aetiological effect of beer drinking. This same problem applies to other correlations involving colorectal cancer, such as those with meat, beef, and fat. In order properly to evaluate the role of beer drinking, it is important that additional, well-designed studies be carried out. Ideally, this means conducting prospective studies of cohorts which have substantially different beer-drinking habits but are similar in all other respects. However, until there are results which show a stronger causal relationship, it seems that correlations and trends involving gross population data may be a rather poor indication of actual aetiology in chronic diseases such as colorectal cancer.

### REFERENCES

AGRICULTURAL RESEARCH SERVICE, U.S. DEPARTMENT OF AGRICULTURE (1956) Food Consumption of Households in the United States, Spring 1955. Washington: U.S. Government Printing Office, Vol. 1–17 and earlier references cited therein.

AGRICULTURAL RESEARCH SERVICE, U.S. DEPARTMENT OF AGRICULTURE (1966) Food Consumption of Households in the United States, Spring 1965. Washington: U.S. Government Printing Office, Vol. 1–18.

ALCOHOL AND HEALTH (1971) First Special Report to the U.S. Congress from the Secretary of Health, Education and Welfare. Washington: DHEW Publ. No. (HSM) 72-9099 (First Printing); 73-9031 (Second Printing).

ALCOHOL AND HEALTH, NEW KNOWLEDGE (1974) Second Special Report to the U.S. Congress from the Secretary of Health, Education and Welfare. Washington: DHEW Publ. No. (ADM) 75–212.

ARMSTRONG, B. & DOLL, R. (1975) Environmental Factors and Cancer Incidence and Mortality in Different Countries, with Special Reference to Dietary Practices. *Int. J. Cancer*, **15**, 617.

BERG, J. W. & HOWELL, M. A. (1974) The Geographic Pathology of Bowel Cancer. *Cancer, Philadelphia*, **34**, 807.

BJELKE, E. (1971) Case-Control Study of the Stomach, Colon, and Rectum. In *Oncology* 1970: *Proc. Tenth Internat. Cancer Congress*. Eds. R. L. Clark, R. C. Cumley, J. E. McCoy and M. M. Copeland. Chicago: Year Book Medical, **5**, 320.

BJELKE, E. (1973) Thesis, University of Minnesota.

BJELKE, E. (1974*a*) Colon Cancer and Blood Cholesterol. *Lancet*, i, 1116.

BJELKE, E. (1974*b*) Personal communication (cited in Breslow and Enstrom, 1974).

BJELKE, E. (1974*c*) Epidemiologic Studies of Cancer of the Stomach, Colon, and Rectum; with Special Emphasis on the Role of Diet. *Scand. J. Gastroenterol.*, **9**, Suppl. 31, 1.

BOYD, J. T. & DOLL, R. (1954) Gastro-intestinal Cancer and the Use of Liquid Paraffin. *Br. J. Cancer*, **8**, 231.

BRESLOW, N. E. & ENSTROM, J. E. (1974) Geographic Correlations Between Cancer Mortality Rates and Alcohol-Tobacco Consumption in the United States. *J. natn. Cancer Inst.*, **53**, 631.

BURBANK, F. (1971) Patterns in Cancer Mortality in the United States: 1950–1967. *Natn. Cancer Inst. Monog.*, **33**, 1.

BUREAU OF THE CENSUS (1973) *Statistical Abstract of the United States*, 1973. Washington: U.S. Government Printing Office.

CUTLER, S. J. (1973) Report on the Third National Cancer Survey: In *Proc. 7th National Cancer Conference*, 1972. Philadelphia: Lippincott.

CUTLER, S. J. & DAVESA, S. S. (1973) Trends in Cancer Incidence and Mortality in the U.S.A. In *Host Environment Interactions on the Etiology of Cancer in Man*. Eds. R. Doll & I. Vodopija. Lyon: International Agency for Research on Cancer.

CUTLER, S. J. & YOUNG, J. L. (1975) Third National Cancer Survey: Incidence Data. *Natn. Cancer Inst. Monog.*, **41**, 1.

DORN, H. F. & CUTLER, S. J. (1959) Morbidity from Cancer in the United States. *Pub. Hlth. Monog.* No. 56.

ENSTROM, J. E. (1975*a*) Colorectal Cancer and Consumption of Beef and Fat. *Br. J. Cancer*, **32**, 432.

ENSTROM, J. E. (1975*b*) Cancer Mortality Among Mormons. *Cancer, Philadelphia*, **36**, 825.

GORI, G. B. (Chairman) (1975) Dietary Factors and Cancer of the Large Bowel, in Symposium on Nutrition in the Causation of Cancer. *Cancer Res.*, **35**, 3388.

GURALNICK, L. (1963) Mortality by Occupational Level and Cause of Death Among Men 20 to 64 Years of Age: United States, 1950. Vital Statistics Special Reports, **53**, No. 5. Washington: U.S. Public Health Service.

HAENSZEL, W., BERG, J. W., SEGI, M., KURIHARA, M. & LOCKE, F. B. (1973) Large-bowel Cancer in Hawaiian Japanese. *J. natn. Cancer Inst.*, **51**, 1975.

HIGGINSON, J. (1966) Etiological Factors in Gastrointestinal Cancer in Man. *J. natn. Cancer Inst.*, **37**, 527.

HOWELL, M. A. (1974) Factor Analysis on International Cancer Mortality Data and *per capita* Food Consumption. *Br. J. Cancer*, **29**, 328.

KETCHAM, A. S., WEXLER, H. & MANTEL, N. (1963) Effects of Alcohol on Mouse Neoplasia. *Cancer Res.*, **23**, 667.

KITAGAWA, E. M. & HAUSER, P. M. (1973) *Differential Mortality in the United States: A Study in Socioeconomic Epidemiology*. Cambridge, Mass.: Harvard University Press.

KLEBBA, A. J., MAURER, J. D. & GLASS, E. J. (1974) Mortality Trends from Leading Causes of Death: United States, 1950–69. Washington: National Center for Health Statistics. DHEW Publ. No. (HRA) 74-1853.

KREBS, C. (1928*a*) *Hospitalstid.*, **71**, 621.

KREBS, C. (1928*b*) *Z. Immun. Exp. Therap.*, **59**, 220.

KURATSUNE, M., KOHCHI, S., HORIE, A. & NISHIZUMI, M. (1971) Test of Alcoholic Beverages and Ethanol Solutions for Carcinogenicity and Tumor-promoting Activity. *Gann*, **62**, 395.

LILIENFELD, A. M., LEVIN, M. L. & KESSLER, I. I. (1972) *Cancer in the United States*. Cambridge: Harvard University Press.

MASON, T. J. & MCKAY, F. W. (1974) U.S. Cancer Mortality by County: 1950–1969. Washington: DHEW Publication No. (NIH) 74–615, U.S. Government Printing Office.

MODAN, B., BARELL, V., LUBIN, F., MODAN, M., GREENBERG, R. A. & GRAHAM, S. (1975) Low-fiber Intake as an Etiologic Agent in Cancer of the Colon. *J. natn. Cancer Inst.*, **55**, 15.

PERNU, J. (1960) An Epidemiological Study on Cancer of the Digestive Organs and Respiratory System. *Ann. Med. Intern. Fenn.*, **49**, Suppl. 33, 1.

PHILLIPS, R. L. (1975) Role of Life-style and Dietary Habits in Risk of Cancer Among Seventh-day Adventists. *Cancer Res.*, **35**, 3513.

RAUNIKAR, R., PURCELL, J. C. & ELROD, J. C. (1973) *Spatial and Temporal Aspects of the Demand for Food in the United States*. Athens: University of Georgia, Vol. 1–10.

STOCKS, P. (1957) Report on Cancer in North Wales and Liverpool Region. In *Br. Emp. Cancer Camp. 35th Annual Report*, Supplement to Part II, p. i.

SUNDBY, P. (1967) *Alcoholism and Mortality*. New Brunswick (N.J.): Rutgers Center on Alcohol Studies, p. 107.

U.S. BREWERS ASSOCIATION, INC. (1972) *Brewing Industry in the United States: Brewers Almanac 1972*. Washington.

WYNDER, E. L. & SHIGEMATSU, T. (1967) Environmental Factors of Cancer of the Colon and Rectum. *Cancer, Philadelphia*, **20**, 1520.

WYNDER, E. L., KAJITANI, T., ISHIKAWA, S., DODO, H. & TAKANO, A. (1969) Environmental Factors of Cancer of the Colon and Rectum: II. Japanese Epidemiological Data. *Cancer, Philadelphia*, **23**, 1210.

YERUSHALMY, J. (1966) On Inferring Causality from Observed Associations. In *Controversy in Internal Medicine*. Eds. F. J. Inzelfinger, A. S. Relman and M. Finland. Philadelphia: W. B. Saunders, p. 659.